# Climate change and marine fisheries: Least developed countries top global index of vulnerability

**DOI:** 10.1371/journal.pone.0179632

**Published:** 2017-06-20

**Authors:** Robert Blasiak, Jessica Spijkers, Kanae Tokunaga, Jeremy Pittman, Nobuyuki Yagi, Henrik Österblom

**Affiliations:** 1 Stockholm Resilience Centre, Stockholm University, Stockholm, Sweden; 2 Graduate School of Agricultural and Life Sciences, The University of Tokyo, Bunkyo-ku, Tokyo, Japan; 3 United Nations University Institute for the Advanced Study of Sustainability, Shibuya-ku, Tokyo, Japan; 4 ARC Centre of Excellence for Coral Reef Studies, James Cook University, Queensland, Australia; 5 Ocean Alliance, The University of Tokyo, Bunkyo-ku, Tokyo, Japan; 6 School of Environment, Resources and Sustainability, University of Waterloo, Waterloo, Canada; Technical University of Denmark, DENMARK

## Abstract

Future impacts of climate change on marine fisheries have the potential to negatively influence a wide range of socio-economic factors, including food security, livelihoods and public health, and even to reshape development trajectories and spark transboundary conflict. Yet there is considerable variability in the vulnerability of countries around the world to these effects. We calculate a vulnerability index of 147 countries by drawing on the most recent data related to the impacts of climate change on marine fisheries. Building on the Intergovernmental Panel on Climate Change framework for vulnerability, we first construct aggregate indices for exposure, sensitivity and adaptive capacity using 12 primary variables. Seven out of the ten most vulnerable countries on the resulting index are Small Island Developing States, and the top quartile of the index includes countries located in Africa (17), Asia (7), North America and the Caribbean (4) and Oceania (8). More than 87% of least developed countries are found within the top half of the vulnerability index, while the bottom half includes all but one of the Organization for Economic Co-operation and Development member states. This is primarily due to the tremendous variation in countries’ adaptive capacity, as no such trends are evident from the exposure or sensitivity indices. A negative correlation exists between vulnerability and per capita carbon emissions, and the clustering of states at different levels of development across the vulnerability index suggests growing barriers to meeting global commitments to reducing inequality, promoting human well-being and ensuring sustainable cities and communities. The index provides a useful tool for prioritizing the allocation of climate finance, as well as activities aimed at capacity building and the transfer of marine technology.

## Introduction

Marine ecosystems and fisheries provide a crucial foundation for human well-being and development, yielding 81.5 million tons of catch in 2014, valued at over USD 90 billion [1]. For many low-income food-deficit countries (LIFDCs) and local communities in developing countries, near-shore marine fisheries also provide a crucial source of micronutrients, which are necessary for early childhood development and influence long-term public health outcomes [2][3]. Simultaneously, demand for fish has been growing, with per capita annual consumption more than doubling since the 1960s, rendering the trade in marine foodstuffs a potential motor for development [1].

Yet sustainable management of marine resources remains a substantial challenge, not least due to continuing uncertainty ranging from the dynamics of fish stocks to the changing biophysical properties of marine systems [4][5]. Climate change is projected to bring manifold impacts to ocean systems, which will influence the life cycles, abundances and distributions of marine species [6][7]. Historically, unpredictable dynamism in fish stocks has posed a serious challenge to fisheries managers, particularly in the case of shared and straddling fish stocks, suggesting that future changes in distribution due to climate regime shifts may result in conflict and negative impacts on local and national economies [8][9][10].

It is highly likely that marine fisheries around the world are vulnerable to the various impacts of climate change. Vulnerability to climate change is defined as the product of three variables, namely: (1) exposure to climate change impacts; (2) sensitivity of an economy/community/country to changes in productive capacity associated with climate change impacts; and (3) adaptive capacity, or the ability to modify or adjust fisheries and livelihoods in order to cope with the negative impacts of climate change and pursue any emerging opportunities [11][12][13]. Allison et al. [14] used this framework to construct a global index of vulnerability of national economies to the impacts of climate change on fisheries, and the approach was further refined with the biophysical models of Barange et al. [15], which consider climate change impacts on fisheries in a sample of 67 countries’ exclusive economic zones (EEZ).

We further refine previous efforts with new and updated data sources relevant to marine capture fisheries. In addition to recalculating the vulnerability index, we highlight the vulnerability of coastal least developed countries (LDCs) and small island developing states (SIDS), and how deficits in adaptive capacity are the primary driver of this vulnerability. To place these findings in a broader policy context, we conclude by referencing global commitments to reduce inequality and promote equitable development, as well as pathways to reducing vulnerability, including by prioritizing the allocation of climate finance and activities to build capacity and promote the transfer of marine technologies.

## Methods

### Refining the vulnerability index

We expand on previous efforts to calculate a global index of the vulnerability of national economies to climate change impacts on fisheries by adding several novel refinements. First, to the greatest possible extent, we disaggregate marine and inland fisheries. This is due not only to the stark differences in management regimes and catch volumes (marine fisheries are roughly seven times as productive as inland fisheries), but also the risks related to assessing both with the same indicator or model [1]. Allison et al. [14] noted the challenge of choosing such a variable, and ultimately opted for mean predicted land surface temperature, while suggesting that sea surface temperature (SST) would likely be the more reliable indicator for impacts on marine fisheries, as it is linked to levels of primary production and the foundations of marine food webs [16]. Their results were therefore somewhat skewed towards countries with substantial inland fisheries and high ratios of land area to EEZ, with land-locked countries occupying seven of the top 20 positions on their vulnerability index [14].

Our second step is therefore to calculate exposure based on SST anomalies within the EEZ of coastal states using CMIP5 multi model ensemble means. While research suggests that warming oceans will result in reduced catches in some regions and larger catches in others [17], sudden changes in either direction can spark conflict [18]. For instance, the spatial distribution and overall abundance of Atlantic mackerel (*Scomber scombrus*) in the northeastern Atlantic expanded from 2008–2014, enabling the scientific Advisory Committee of the International Council for the Exploration of the Sea (ICES) to nearly double recommended total allowable catch (TAC) levels from around 500,000 tons to over 1 million tons [19]. Yet conflict erupted due to singleton behavior, when individual actors broke with the existing coalition, leading to retaliatory actions and ultimately the TAC levels being exceeded [20][18]. Even in such closely monitored commercial fisheries, different modeling approaches result in vastly different projections of habitat suitability and abundance levels [21][4].

The internal variability of climate change models increases at smaller spatial scales, rendering projections more effective at a macro level than a species-specific one [22]. Furthermore, the unpredictability of how socio-economic factors included in this analysis will be affected by the impacts of climate change on fisheries, and corresponding feedback loops, creates a high degree of scenario uncertainty, suggesting that our analysis should be limited to a relatively short time horizon [23]. Still, accounting for natural variability, particularly in areas like the North Pacific, suggests the need for projections that extend beyond a decadal timeframe [24]. We therefore depart from previous long-term efforts to forecast vulnerability to the impacts of climate change, and instead limit our projection to the near future from 2016–2050. To allow comparability with longer-term future projections, however, we also include supplemental material with projections over a similar 35-year timeframe from 2066–2100.

Third, we revisit the constructed variable for adaptive capacity in Allison et al. [14], which is based on four broad socio-economic variables: healthy life expectancy; education (literacy rates and school enrolment ratios); governance (including political stability); size of economy (GDP). None of these factors, however, are specifically related to the fisheries sector, but rather they’re relevant for the adaptive capacity of any sector within a given country in the face of any type of stress or disaster. One strength of selecting such broad socio-economic variables is that they may reflect the mobility of those employed in the fisheries sector and their capacity to move into other sectors of the economy as needed. However, these indicators do not provide fisheries-specific information, and it is uncertain whether fishers or the fisheries sector are able to draw on the capacities suggested by these indicators.

We attempt to improve the adaptive capacity calculation by including additional factors that are directly tied to the fisheries sector. We incorporate the proportion of industrial and small-scale fishers to the adaptive capacity calculation. Industrialized fisheries typically have greater access to technology and markets than small-scale fisheries, and they are more mobile–all factors that have been shown to enhance adaptation to climate-related stressors [25][26]. We include latest estimated percentages of industrial/small-scale fisheries from the Sea Around Us Project [27], and we assume that higher proportions of industrial fishers increase adaptive capacity. Likewise, fisheries subsidies can provide a cushion to the sector within the context of inter-annual variability in catch levels or sudden disturbances. Such subsidies are moreover directly tied to the fisheries sector, and could broadly be considered as a proxy for the capacity or willingness of governments to assist the sector in resolving new challenges. We therefore supplement the adaptive capacity variable with the inclusion of fisheries subsidy data from the Sea Around Us Project. [28][29]

Finally, in the case that variables used in the original index were retained, each was updated to reflect the most recent available data. Since the most recent data used by Allison et al. [14] comes from 2001, in most cases the updating process constituted a decade or more of new data, as outlined in the subsequent section.

### Recalculating the vulnerability index

To facilitate broad comparability with past literature, the index is built in line with the IPCC framework that vulnerability (*V*) is a function of exposure (*E*), sensitivity (*S*) and adaptive capacity (*AC*) (1). Indices of exposure, sensitivity and adaptive capacity were first assembled in line with the methodology of Allison et al. [14] with some modifications as described in the following sections. As with the original methodology, variables were normalized (indicated with a subscript *N*) on a scale from 0 to 1, and subsequent component indices were likewise normalized. The calculation of the component indices for exposure, sensitivity and adaptive capacity is given in Eqs (2), (3) and (4).

$$V=E_{N}+S_{N}+AC_{N}$$ (1)

$$E=E_{1N}$$ (2)

$$S=Average(S_{1N}+S_{2N}+S_{3N}+S_{4N}+S_{5N})$$ (3)

$$AC=0.5\;*\;[Average(AC_{1N}+AC_{2N})+Average(AC_{3N}+AC_{4N}+AC_{5N}+AC_{6N})]$$ (4)

**Fig 1:**
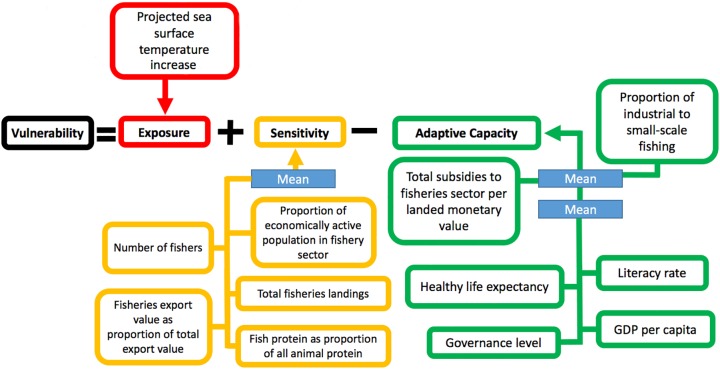
Overview of variable construction and calculation of vulnerability index.

The individual variables used in these equations are as follows. For exposure (*E*), projected sea surface temperature anomalies (*E*_*1*_) are calculated for different timeframes and representative concentration pathways. The variables for sensitivity (*S*) are: number of fishers (*S*_*1*_); share of marine fisheries exports in total exports (*S*_*2*_); percentage of fishers in the economically active population (*S*_*3*_); weight of total fisheries landings (*S*_*4*_); and share of marine fish protein in total protein consumption (*S*_*5*_). The variables for adaptive capacity (*AC*) are: fisheries subsidies (*AC*_*1*_); ratio of industrial to small-scale fisheries (*AC*_*2*_); healthy life expectancy (*AC*_*3*_); governance capacity (*AC*_*4*_); education levels (*AC*_*5*_); per capita gross domestic product (GDP) (*AC*_*6*_). A graphical representation of the index is available in Fig 1.

#### Exposure

**Fig 2:**
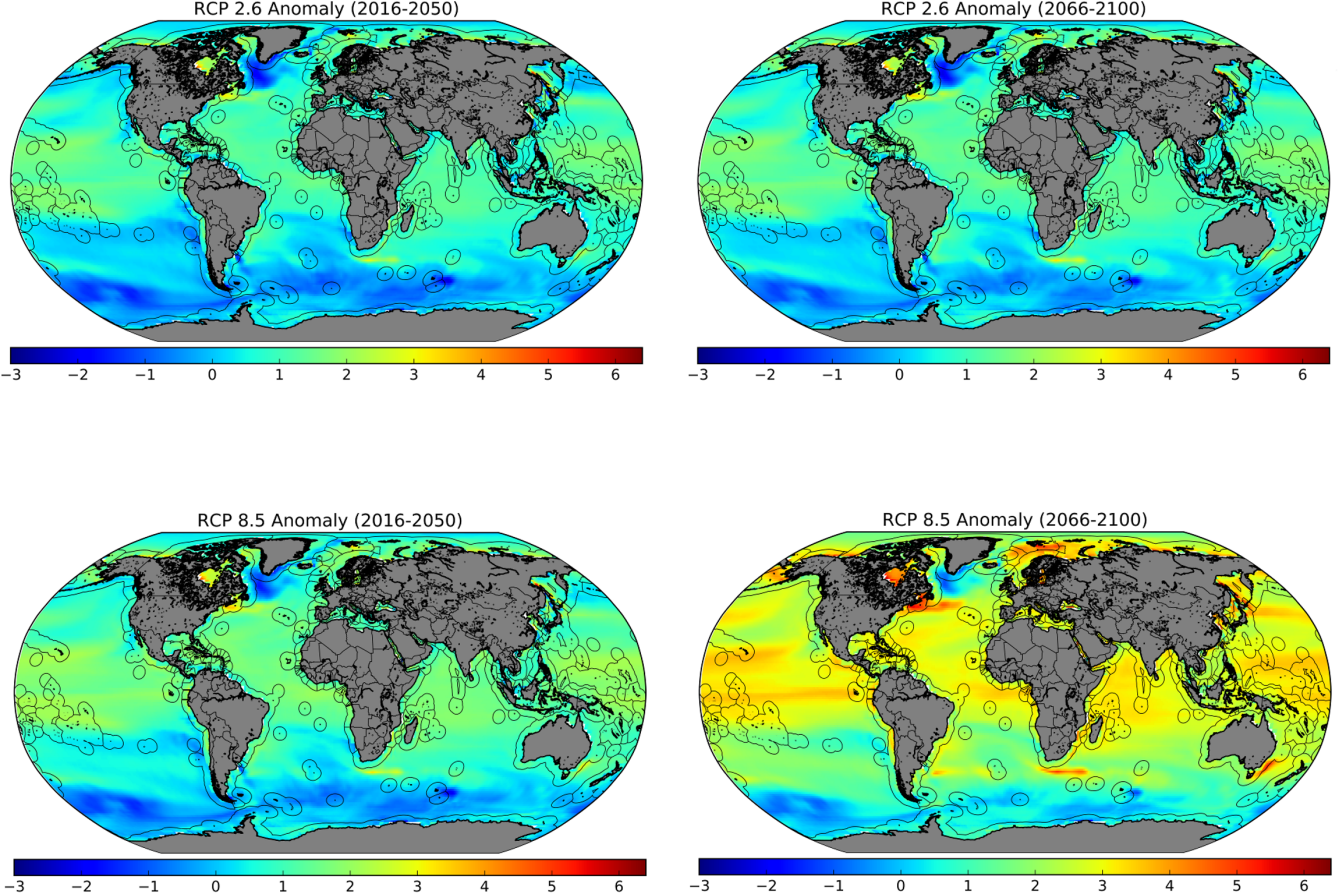
Average annual sea surface temperature anomalies at representative concentration pathways 2.6 and 8.5 for two different 35-year timeframes (2016–2050; 2066–2100) compared with a reference climatology (1900–1950).

The exposure of countries to the impacts of climate change on marine capture fisheries was calculated based on projected sea surface temperature anomalies. Three different representative concentration pathways (RCPs) were used to provide insight into exposure levels in the case of highly successful reduction of greenhouse gas emissions (RCP 2.6 –highly optimistic), more modest emissions reductions (RCP 4.5 –optimistic), and a continued increase in carbon emissions (RCP 8.5 –closer to business-as-usual, somewhat pessimistic). Multi-model ensemble means were constructed using data from all CMIP5 models with outputs that satisfied three criteria: (1) available for RCPs 2.6, 4.5 and 8.6; (2) available for the projected timeframe from 2016–2100, as well as a historical reference timeframe from 1900–1950; (3) including the variable sea surface temperature (“tos”). See S1 Appendix for a list of the 14 models used to calculate the multi-model ensemble means. Output data files for each model were downloaded from the Earth System Grid Federation [30], concatenated using NetCDF command line operators, regridded from native grids to a standardized rectangular grid, and averaged for all 14 models. The same process was used to construct four NetCDF (Network Common Data Form) files, namely projected sea surface temperature (SST) from 2016–2100 at RCP 2.6, RCP 4.5 and RCP 8.5, as well as a reference climatology from 1900–1950. Finally, average SST anomalies for each of the three RCPs were computed for two future time periods, a near-future projection (2016–2050) and a distant-future projection (2066–2100) by calculating the average SST over each respective timeframe, and then subtracting the average SST from the reference climatology (1900–1950). The results for both the near-future and distant-future projections at RCP 2.6 and RCP 8.5 are presented in Fig 2 with an overlayed shapefile of the exclusive economic zones (EEZs).

Next, the exposure of each country to SST anomalies was calculated for each of the three pathways as well as the two timeframes. For cases in which a country’s EEZ was divided into multiple polygons in the EEZ shapefile [31], these were first combined according to the sovereignty feature. The points of each polygon were used to determine which grid points to extract from the SST anomaly files. These points were subsequently averaged to give the SST anomaly for each country, resulting in a total of six indices (three RCPs with two timeframes each). Finally, each index was normalized on a scale from 0 to 1. Unix and Python scripts used for the manipulation of CMIP5 files and the subsequent SST anomaly calculations are available from Kristiansen [32][33] and were adapted to the needed timeframes, climate variables and marine regions.

#### Sensitivity

The sensitivity or dependence of countries with regard to marine capture fisheries was calculated as an index of five variables: number of fishers (*S*_*1*_); share of marine fisheries exports in total exports (*S*_*2*_); percentage of fishers in the economically active population (*S*_*3*_); weight of total fisheries landings (*S*_*4*_); and share of marine fish protein in total protein consumption (*S*_*5*_) (Fig 1). In line with the previous work of Allison et al. [14], values for each variable were first collected from corresponding existing datasets. Next the data were normalized on a scale from 0 to 1, and the five variables were averaged with equal weights. The subsequent aggregate sensitivity variable was then normalized on a scale from 0 to 1 to create the sensitivity index.

FAO reports data on number of fishers, but the level of detail varies across countries, with some going so far to disaggregate according to gender and deep sea / marine / inland fishing, while others provide aggregate data [1][34]. Whenever possible, the latest disaggregated data was used for this analysis, but the authors acknowledge the variation in data quality as a limitation. As previously noted, there is only a weak correlation between the number of fishers per country and the proportion of the economically active population that are fishers [14][35]; we found that this trend continues to persist (Spearman’s *ρ* = 0.28) and therefore included both variables in our sensitivity index. The share of fisheries exports in total exports was calculated by dividing each country’s total exports by the latest FAO fisheries commodities trade flow data [36][37]. Due to interannual variability in fisheries catch volumes, total fisheries landings were calculated as the mean of catches from 2012–2014 [38]. The share of marine fish protein in total protein consumption was calculated as a percentage using the latest values available on individual food balance sheets [39].

#### Adaptive capacity

Six variables, including two constructed from multiple sub-variables, were combined to calculate the index of adaptive capacity: fisheries subsidies (*AC*_*1*_); ratio of industrial to small-scale fisheries (*AC*_*2*_); healthy life expectancy (*AC*_*3*_); governance capacity (unweighted mean of six dimensions of governance) (*AC*_*4*_); education levels (combination of literacy rates and school enrolment rates) (*AC*_*5*_); per capita gross domestic product (GDP) (*AC*_*6*_) (Fig 1). Total fisheries subsidies as a percentage of landed monetary value were collected from the Sea Around Us project, averaged for available years, and normalized [28][29]. The ratio of industrial to small-scale (artisanal, subsistence and recreational) fisheries (in 2010) was likewise compiled from the latest Sea Around Us data [27]. Statistics from 2015 on healthy life expectancy (HALE) at birth were collected and normalized [40]. Due to the exceptionally high degree of correlation between literacy rates and primary school enrolment rates for countries reporting both types of data (Spearman’s *ρ* = 0.97), these values were used interchangeably to fill in gaps in each respective dataset to remove the necessity of excluding countries from the analysis [41][42]. The Worldwide Governance Indicators project calculates governance across six individual dimensions, namely voice and accountability, political stability and absence of violence, government effectiveness, regulatory quality, rule of law, and control of corruption; we calculated an unweighted average of these six individual values and normalized this average to generate a single governance variable [43]. Finally, the latest per capita GDP levels were extracted from the World Bank Development Indicators database [44]. While the calculation of the adaptive capacity index starts with the same method as the sensitivity index described in 2.2, a further step was taken, namely the weighting of the two fisheries-related variables. This departure from the methodology of Allison et al. [14] is meant to ensure that all three elements of the vulnerability index (exposure, sensitivity and adaptive capacity) are explicitly linked to the marine context. The adaptive capacity variables used by Allison et al. [14] are all broad socio-economic factors without a direct connection to the fisheries sector. The mean of these four normalized socio-economic variables makes up one half of the adaptive capacity index, while the mean of the two normalized fisheries-related variables makes up the second half of the adaptive capacity index (Fig 1). This gives the fisheries-related variables a greater weight than the socio-economic variables, and results in an adaptive capacity index more specifically designed to match the fisheries sector. As with the sensitivity index, the resulting adaptive capacity index is then normalized on a scale from 0 to 1.

Landlocked countries were not considered in this analysis, and a lack of data led to the exclusion of an additional 19 countries (S2 Appendix), resulting in an index of 147 countries (S1 Table). Finally, a vulnerability index was calculated in line with the IPCC framework (exposure + sensitivity–adaptive capacity = vulnerability), and the resulting vulnerability index was normalized on a scale from 0 to 1 (Table 1). While a total of six separate vulnerability indices are calculated for RCPs 2.6 4.5 and 8.5 under the two timeframes (2016–2050, 2066–2100), and are available in S1 Table, the remainder of paper focuses on the vulnerability index for RCP 8.5 over the timeframe 2016–2050.

**Table 1:** National vulnerability to the impacts of climate change on marine fisheries.

First quartile			Second quartile			Third quartile				Fourth quartile		
Rank	Country	Vulnerability Score	Rank	Country	Vulnerability Score	Rank	Country		Vulnerability Score	Rank	Country	Vulnerability Score
1	KIRIBATI	0.999999999	38	SOMALIA	0.527489935	75	CONGO, DEM. REP.	0.426384127		112	COLOMBIA	0.295724865
2	MICRONESIA, FED. STS.	0.909266359	39	LEBANON	0.526767923	76	NICARAGUA	0.42159378		113	MALTA	0.295083491
3	SOLOMON ISLANDS	0.901230309	40	GUINEA	0.521582999	77	GUATEMALA	0.416361127		114	CAMBODIA	0.293324132
4	MALDIVES	0.867723508	41	KENYA	0.518308334	78	CUBA	0.413576655		115	KOREA, DEM. REP.	0.285808758
5	VANUATU	0.818550262	42	JORDAN	0.517966676	79	GREECE	0.413243596		116	NORWAY	0.28470084
6	SAMOA	0.810912605	43	VIETNAM	0.514346424	80	BRAZIL	0.412039257		117	CROATIA	0.282781684
7	MOZAMBIQUE	0.809247649	44	VENEZUELA, RB	0.513516643	81	EQUATORIAL GUINEA	0.408156022		118	PANAMA	0.276858295
8	CHINA	0.765473303	45	DOMINICA	0.510738028	82	SAUDI ARABIA	0.403629959		119	LITHUANIA	0.27658499
9	SIERRA LEONE	0.752615175	46	GUYANA	0.506518939	83	MOROCCO	0.400028173		120	GEORGIA	0.275699519
10	TUVALU	0.717797193	47	HONDURAS	0.501765408	84	MAURITIUS	0.398363489		121	ISRAEL	0.274817835
11	HAITI	0.699963225	48	RUSSIAN FEDERATION	0.501432395	85	ECUADOR	0.396644315		122	TURKEY	0.263301033
12	BENIN	0.688742195	49	GRENADA	0.500578354	86	BARBADOS	0.39549722		123	ALBANIA	0.261815531
13	SÃO TOMÉ AND PRINCIPE	0.675062392	50	TANZANIA	0.497444379	87	TRINIDAD AND TOBAGO	0.394045416		124	ARUBA	0.257629784
14	COMOROS	0.674417081	51	TOGO	0.496878553	88	PHILIPPINES	0.393650591		125	ITALY	0.255898034
15	NIGERIA	0.647307005	52	ANTIGUA AND BARBUDA	0.493384448	89	ST. LUCIA	0.393483814		126	FINLAND	0.255118645
16	GHANA	0.635472868	53	ERITREA	0.491403911	90	CYPRUS	0.377404848		127	BULGARIA	0.252121525
17	CAMEROON	0.629894742	54	CANADA	0.490642638	91	ALGERIA	0.372478186		128	GERMANY	0.242783003
18	BANGLADESH	0.627542039	55	SYRIAN ARAB REPUBLIC	0.487092341	92	IRAN, ISLAMIC REP.	0.368042494		129	KOREA, REP.	0.240988697
19	MADAGASCAR	0.614579906	56	BAHRAIN	0.483456871	93	PAPUA NEW GUINEA	0.367819471		130	SOUTH AFRICA	0.238938298
20	TONGA	0.611863419	57	ST. VINCENT AND THE GRENADINES	0.478559473	94	ROMANIA	0.365042589		131	POLAND	0.235972868
21	BELIZE	0.607443847	58	DOMINICAN REPUBLIC	0.478365168	95	QATAR	0.364826164		132	SLOVENIA	0.222194887
22	CÔTE D'IVOIRE	0.604657435	59	LIBYA	0.47780711	96	SINGAPORE	0.357804744		133	AUSTRALIA	0.220030046
23	SENEGAL	0.60286265	60	PAKISTAN	0.475455416	97	KUWAIT	0.351118519		134	DENMARK	0.216633221
24	GUINEA-BISSAU	0.602297241	61	CAPE VERDE	0.466089281	98	TUNISIA	0.348092428		135	BELGIUM	0.214749952
25	YEMEN, REP.	0.600418482	62	BAHAMAS, THE	0.464906157	99	MONTENEGRO	0.345855903		136	SPAIN	0.206117683
26	INDONESIA	0.594090877	63	MAURITANIA	0.456020801	100	FRANCE	0.345555062		137	JAPAN	0.203742323
27	FIJI	0.589598212	64	GABON	0.454549991	101	BRUNEI DARUSSALAM	0.344607474		138	NETHERLANDS	0.176120438
28	SEYCHELLES	0.585155228	65	DJIBOUTI	0.451695056	102	EGYPT, ARAB REP.	0.344532488		139	ARGENTINA	0.167003997
29	INDIA	0.582425245	66	LIBERIA	0.451286962	103	COSTA RICA	0.328211161		140	SWEDEN	0.16346815
30	ST. KITTS AND NEVIS	0.564704777	67	UNITED ARAB EMIRATES	0.448962558	104	ESTONIA	0.32544234		141	URUGUAY	0.15826322
31	SUDAN	0.561655443	68	MYANMAR	0.446662532	105	UKRAINE	0.323169487		142	UNITED STATES	0.15728165
32	GAMBIA, THE	0.55890093	69	MACAO SAR, CHINA	0.444714953	106	MALAYSIA	0.322866129		143	NAMIBIA	0.156395105
33	TIMOR-LESTE	0.553250138	70	CONGO, REP.	0.444645336	107	PERU	0.322511633		144	ICELAND	0.151805576
34	JAMAICA	0.546889343	71	EL SALVADOR	0.439020018	108	PORTUGAL	0.311161852		145	UNITED KINGDOM	0.12728723
35	SRI LANKA	0.528850589	72	SURINAME	0.431840825	109	HONG KONG SAR, CHINA	0.306846496		146	CHILE	0.118632105
36	ANGOLA	0.528329802	73	THAILAND	0.429072042	110	LATVIA	0.302593139		147	IRELAND	0.102390556
37	KIRIBATI	0.605730263	74	OMAN	0.493023399	111	MEXICO	0.340467051				

## Results

The countries most vulnerable to the effects of climate change on fisheries are primarily small island states in the Pacific Ocean and Caribbean, and countries along the Western and Eastern coasts of Africa (Table 1). Seven of the top ten positions on the index are held by small island developing states (SIDS), while states with substantial inland fisheries such as Tanzania and Cambodia occupied lower rankings than in previous studies (Table 1). Likewise, the vulnerability index calculated by Barange et al. [15] using coupled physical-biological shelf seas models across 67 EEZs matches closely with our findings with the notable exception of the Russian Federation, which occupies a position on the lower half of the vulnerability index. This is perhaps due to the Russian Federation’s high levels of fisheries subsidies and emphasis on industrial fishing, which accounted for over 90% of landed catch in 2010 [27].

Vulnerability of national economies to climate change impacts on fisheries is strongly linked to states’ respective levels of development. All 31 of the world’s least developed countries (LDCs) with coastlines are included in the calculated vulnerability index, and over 87% are in the top half of the vulnerability index (Table 2). Likewise, 29 OECD member states are coastal, and 25 of these are in the bottom half of the index. The individual component indices, however, show greater variation, with both LDCs and OECD states fairly equally distributed across the index of exposure, and slight tendencies to greater sensitivity among LDCs and less sensitivity among OECD states. The adaptive capacity of the two groups of states are a virtual mirror image, with nearly all LDCs appearing on the lower half of the index, while almost all OECD states appear in the upper half of the index (with 86% in the top quartile) (Table 2).

**Table 2:** Overview of economic groupings across the vulnerability index (E: exposure; S: sensitivity; AC: adaptive capacity; V: vulnerability).

	Least Developed Countries				Organization for Economic Cooperation and Development (OECD) member states			
	E	S	AC	V	E	S	AC	V
**1**^**st**^ **Quartile**	11	11	0	**18**	11	4	25	**0**
**2**^**nd**^ **Quartile**	5	9	5	**9**	4	5	2	**4**
**3**^**rd**^ **Quartile**	9	4	8	**3**	4	15	2	**9**
**4**^**th**^ **Quartile**	6	7	18	**1**	10	5	0	**16**

Some geographical regions are also particularly well-represented across the different quartiles of the vulnerability index (Table 3). All 29 countries in Europe, for instance, appeared in the lower two quartiles of the index. The highest quartile is dominated by countries in Africa (17), in addition to small island states in North America and the Caribbean (4), Asia (7) and Oceania (8).

**Table 3:** Geographical distribution of vulnerability.

	Africa	Asia	Europe	North America and the Caribbean	Oceania	South America
**1**^**st**^ **Quartile**	17	7	0	4	8	0
**2**^**nd**^ **Quartile**	13	12	0	9	0	3
**3**^**rd**^ **Quartile**	6	9	9	8	1	4
**4**^**th**^ **Quartile**	2	6	20	3	2	3
**Totals**	**38**	**34**	**29**	**24**	**11**	**10**

Considering multiple representative concentration pathways (RCPs) as well as two different 35-year timescales (2016–2050; 2066–2100) provided insight into how climate change mitigation efforts at the global level could affect the relative vulnerability of individual countries. Full indices are calculated for all three RCPs on the two timescales (S1 Table), and the five most/least vulnerable countries for each are listed in Table 4. Although there is some movement of countries up or down the index, the same trend remains of SIDS occupying the top of the index, and OECD member states consistently remaining among the least vulnerable countries.

**Table 4:** Five most vulnerable and five least vulnerable countries across different representative concentration pathways (RCPs) and timeframes.

Near-future scenario (2016–2050)				Distant-future scenario (2066–2100)		
RCP 2.6	RCP 4.5	RCP 8.5	(Rank)	RCP 2.6	RCP 4.5	RCP 8.5
KIRIBATI*	KIRIBATI*	KIRIBATI*	1	KIRIBATI*	KIRIBATI*	KIRIBATI*
MALDIVES*	MALDIVES*	MICRONESIA, FED. STATES*	2	MALDIVES*	MOZAMBIQUE	SOLOMON ISLANDS*
SOLOMON ISLANDS*	SOLOMON ISLANDS*	SOLOMON ISLANDS*	3	SOLOMON ISLANDS*	SIERRA LEONE	TUVALU*
MICRONESIA, FED. STATES*	SIERRA LEONE	MALDIVES*	4	MICRONESIA, FED. STATES*	SAMOA*	VANUATU*
MOZAMBIQUE	MICRONESIA, FED. STATES*	VANUATU*	5	SIERRA LEONE	COMOROS*	MALDIVES*
…	…	…	…	…	…	…
NAMIBIA	CHILE**	ICELAND**	143	IRELAND**	NAMIBIA	CHILE**
CHILE**	UNITED KINGDOM**	UNITED KINGDOM**	144	UNITED KINGDOM**	NETHERLANDS**	NEW ZEALAND**
IRELAND**	NAMIBIA	CHILE**	145	NAMIBIA	IRELAND**	UNITED KINGDOM**
ARGENTINA	IRELAND**	IRELAND**	146	ARGENTINA	UNITED KINGDOM**	ARGENTINA
NEW ZEALAND**	NEW ZEALAND**	NEW ZEALAND**	147	NEW ZEALAND**	ICELAND**	AUSTRALIA**

(* = small island developing states (SIDS)

** = members of the Organization for Economic Co-operation and Development (OECD))

## Discussion

Covering EEZs of 147 countries, this is the most comprehensive assessment to date of vulnerability due to climate change impacts on marine fisheries. The index reflects clear trends with regards to countries’ level of development, and the disaggregation of vulnerability into exposure, sensitivity and adaptive capacity provides some insight into how to increase social-ecological resilience in LDCs and SIDS. Most notably, no linkage is apparent between exposure to the impacts of climate change on fisheries and national development levels, with both LDCs and OECD member states spread across the exposure index. This can be interpreted as an empowering finding, because pathways to reducing exposure to the impacts of climate change will require global action and long, uncertain timeframes. Adaptive capacity, on the other hand, can be enhanced in a more direct and timely fashion through action at national and sub-national levels, and further supported by regional and global partnerships. Changes in sensitivity can also affect the overall vulnerability score, yet while a reduction in exposure or increase in adaptive capacity may find broad acceptance, a reduction in sensitivity is less clear cut. This could entail, for instance, a reduction in the number of fishers (perhaps leading to loss of livelihoods and greater unemployment–or movement into other sectors, like agriculture, that may be even more vulnerable to climate change), a reduction in total fisheries landings (perhaps leading to a loss in revenue), or a reduction in fish protein as proportion of all animal protein (perhaps leading to negative health outcomes). Accordingly, the low vulnerability scores of OECD states are also attributable in part to a continuous drop in employment in the fisheries sector over the past decades, as well as the economic importance of the fisheries sector shrinking in comparison with the overall economy [1]. Although perhaps slow to bear fruit, efforts to bolster adaptive capacity constitute the least contentious and most feasible option for countries to reduce vulnerability levels, particularly when considered over longer timeframes like 2016–2050.

Regional heterogeneity with regards to vulnerability has been a source of conflict in some transboundary natural resource management scenarios [45][46][47][48], suggesting that regions with the greatest variability in vulnerability scores may be particularly conflict-prone if exposure to the impacts of climate change on fisheries becomes more pronounced without mitigating efforts to build adaptive capacity. A number of general principles are emerging from multiple case studies that described key factors that contribute to building social-ecological resilience to shocks and disturbances [49], including maintaining diversity and redundancy, managing connectivity, encouraging learning and promoting polycentric governance. Translating these principles into practice involves strengthening existing institutions in the most vulnerable countries identified here, and managing issues that further increase their vulnerability. As described above, such efforts may prove contentious. Providing fishing subsidies or encouraging industrial fishing of certain stocks to promote efficiency, for instance, could result in long-term decreases in national vulnerability scores, but immediately destabilize local fishing communities, leading to negative impacts on local livelihoods, employment, nutrition and ecosystems. Clearly defined interventions that take a long-term holistic approach including local, regional and national levels incorporating both social and ecological goals are therefore crucially important.

While the sensitivity component of this analysis reveals hotspots of SST anomalies under different RCPs, which will likely impact fisheries and their management, it does not attempt to quantify these impacts. A potentially useful further step for improving the index would be to specify projected changes in fisheries productivity, for instance with population models that incorporate not only SST anomalies, but also other factors such as projected changes in primary production or acidity levels [50]. Additionally, the homogenizing effect of averaging SST anomalies across EEZs will disproportionately affect countries with EEZs characterized by high levels of latitudinal variation. Yet similar homogenization is reflected in the socio-economic variables used to calculate the sensitivity and adaptive capacity indices (e.g. GDP per capita, fish protein as a proportion of all animal protein), which provide general estimates and enable comparability across nations, but mask regional or local variabilities. Thus, further refinements in the calculation of the exposure index would optimally be accompanied by refinements to the calculation of socio-economic variables, or would need to be undertaken at local or regional level [15][51].

Initial studies have suggested that catch potential will sharply decrease in some regions, while increasing in others under different climate scenarios [15][17]. Adapting to such changes will require effective fisheries management, and a recent analysis [52] suggested that China, Indonesia and India represent the three countries where there is most potential for improvement in fisheries management. All three countries were identified in the first quartile of the vulnerability index constructed in this study. Although Paragraph XI of the UN Convention on the Law of the Sea (UNCLOS) specifically describes the need for capacity building and the transfer of marine technology, these obligations by the 157 signatories to UNCLOS have been cited as the least implemented section of the convention [53]. Yet the results of this study clearly indicate where capacity building has the potential to represent a key strategy for improving fisheries management and strengthening particularly vulnerable states.

**Fig 3:**
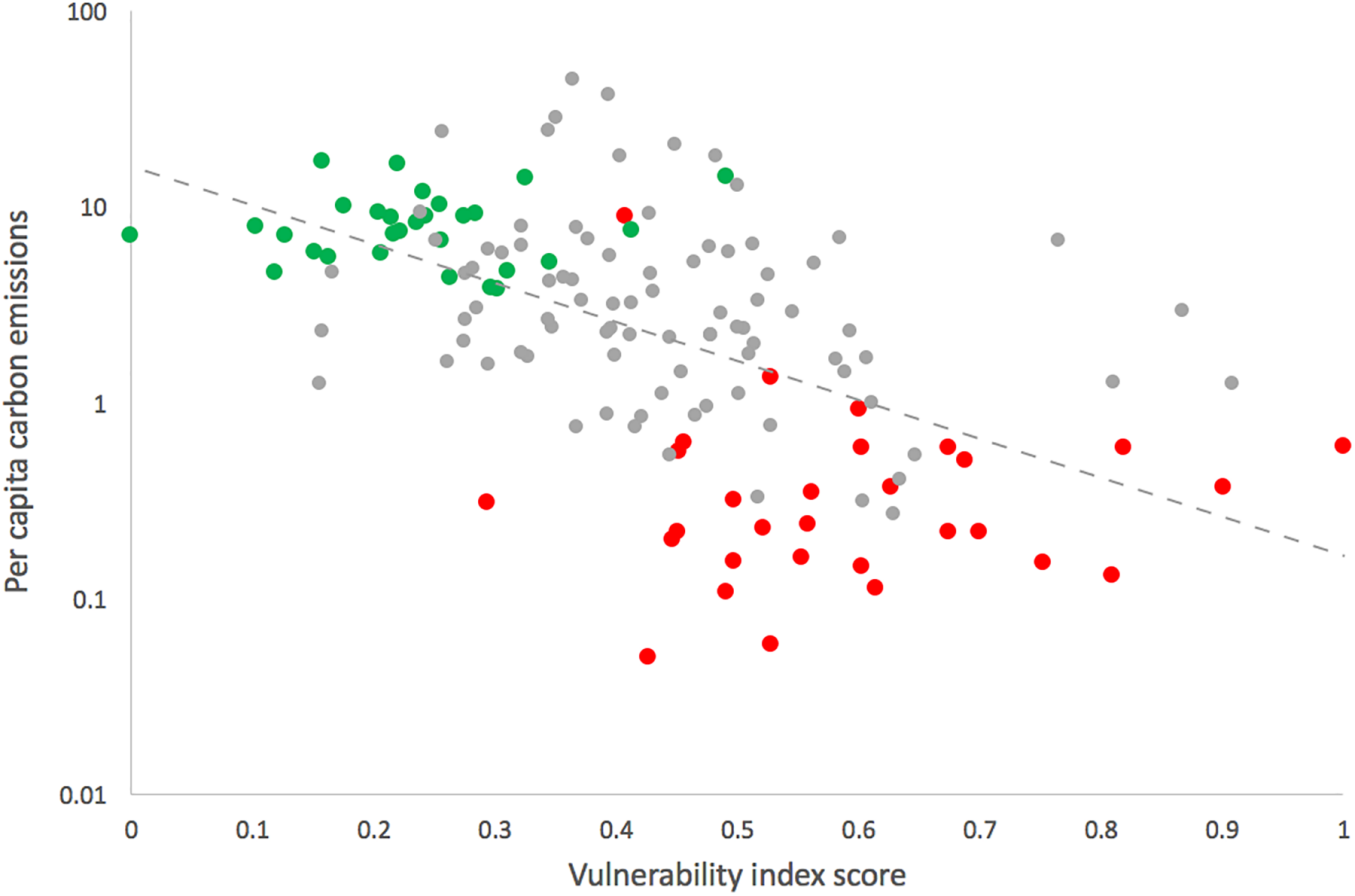
Negative correlation between per capita carbon emissions (metric tons per capita) and states’ vulnerability to the impacts of climate change on fisheries (Spearman’s ρ = -0.60, R^2^ = 0.300, p < 0.0001). Red points indicate Least Developed Countries (LDCs), and green points indicate Organization for Economic Co-operation and Development (OECD) member states. The remaining grey points are neither OECD states nor LDCs.

Previous work has indicated that, historically, highly industrialized countries have contributed a larger per capita share of global carbon emissions than less developed countries, and in many cases continue to do so [54][55]. Likewise, a comparison of our constructed vulnerability index with per capita carbon emissions [56] finds a negative correlation (Pearson’s *ρ* = -0.60, *R*^*2*^ = 0.300, *p* < 0.0001). LDCs occupy the upper range of the vulnerability index, while also having some of the lowest per capita carbon emissions (Fig 3).

There is a substantial body of international commitments and agreements to reduce regional and global inequalities, promote responsible consumption and production patterns, and establish sustainable cities and communities, among other things [57][58]. As this research underscores, systematically reducing vulnerability to the impacts of climate change on marine fisheries is closely linked to development objectives. One possible avenue for translating these commitments into tangible actions is the rapidly expanding pool of financing available through climate funds, which is now estimated at up to USD 650 billion annually for all countries [59]. Dedicated financing has also been rising, with over USD 9 billion approved for new projects in 2014 –nearly double 2012 levels [60]. Fisheries exist at the nexus of food security, income generation and natural resource management, and the earmarking of climate finance to enhance adaptive capacity in countries scoring highly on the vulnerability index could bring a wide range of benefits.

## Supporting information

S1 Table Country data and classifications for calculating vulnerability indices at different representative concentration pathways. (XLSX)

S1 Appendix List of CMIP5 models used to calculate multi-model ensemble means. CanESM2, CNRM-CM5, GFDL-CM3, GFDL-ESM2G, GFDL-ESM2M, GISS-E2-H, GISS-E2-R, HadGEM2-AO, MIROC-ESM-CHEM, MIROC5, MPI-ESM-LR, MPI-ESM-MR, NorESM1-M, NorESM1-ME.
(DOCX)

S2 Appendix List of countries excluded from index due to lack of data. American Samoa, Anguilla, Bermuda, Cayman Islands, Cook Islands, Faroe Islands, French Guiana, Greenland, Guam, Marshall Islands, Martinique, Monaco, Nauru, Netherlands Antilles, New Caledonia, Niue, Palau, Reunion, Taiwan.
(DOCX)
