# Supplementary material for: Climate change and marine fisheries: Least developed countries top global index of vulnerability
Source: PLoS One. 2017 Jun 20;12(6):e0179632. doi: 10.1371/journal.pone.0179632 (PMC5478141; doi:10.1371/journal.pone.0179632)
Supplement: S1 Appendix — CanESM2, CNRM-CM5, GFDL-CM3, GFDL-ESM2G, GFDL-ESM2M, GISS-E2-H, GISS-E2-R, HadGEM2-AO, MIROC-ESM-CHEM, MIROC5, MPI-ESM-LR, MPI-ESM-MR, NorESM1-M, NorESM1-ME. (DOCX) [file pone.0179632.s002.docx]

**S1 Appendix: List of CMIP5 models used to calculate multi-model ensemble means**

CanESM2, CNRM-CM5, GFDL-CM3, GFDL-ESM2G, GFDL-ESM2M, GISS-E2-H, GISS-E2-R, HadGEM2-AO, MIROC-ESM-CHEM, MIROC5, MPI-ESM-LR, MPI-ESM-MR, NorESM1-M, NorESM1-ME
