# Supplementary material for: Climate change and marine fisheries: Least developed countries top global index of vulnerability
Source: PLoS One. 2017 Jun 20;12(6):e0179632. doi: 10.1371/journal.pone.0179632 (PMC5478141; doi:10.1371/journal.pone.0179632)
Supplement: S2 Appendix — American Samoa, Anguilla, Bermuda, Cayman Islands, Cook Islands, Faroe Islands, French Guiana, Greenland, Guam, Marshall Islands, Martinique, Monaco, Nauru, Netherlands Antilles, New Caledonia, Niue, Palau, Reunion, Taiwan. (DOCX) [file pone.0179632.s003.docx]

**S2 Appendix: List of countries excluded from index due to lack of data**

American Samoa, Anguilla, Bermuda, Cayman Islands, Cook Islands, Faroe Islands, French Guiana, Greenland, Guam, Marshall Islands, Martinique, Monaco, Nauru, Netherlands Antilles, New Caledonia, Niue, Palau, Reunion, Taiwan.
